# Increased Anxiety and Depression Among Belgian Sexual Minority Groups During the First COVID-19 Lockdown—Results From an Online Survey

**DOI:** 10.3389/fpubh.2022.797093

**Published:** 2022-04-11

**Authors:** Thijs Reyniers, Veerle Buffel, Estrelle Thunnissen, Bea Vuylsteke, Magdalena Siegel, Christiana Nöstlinger, Edwin Wouters

**Affiliations:** ^1^Department of Public Health, Institute of Tropical Medicine, Antwerp, Belgium; ^2^Department of Sociology, Centre for Population, Family and Health University of Antwerp, Antwerp, Belgium; ^3^Department of Developmental and Educational Psychology, University of Vienna, Vienna, Austria; ^4^Centre for Health Systems Research & Development, University of the Free State, South Africa

**Keywords:** depression, LGBT, social contact, COVID-19, physical distancing, anxiety, lockdown

## Abstract

The COVID-19 pandemic most likely had a negative impact on mental health. Sexual minorities are at higher risk for adverse mental outcomes such as depression, anxiety and suicidal ideation. Such mental health disparities may have exacerbated during the COVID-19 pandemic, due to restricted real-life social contact. The study aim was to examine changes in depression, anxiety and suicidal ideation among Belgian sexual minority adults between the periods before and during the first COVID-19 lockdown. We conducted an online survey, which was disseminated by community organizations throughout Belgium in April 2020. The questionnaire included two-item Generalized-Anxiety-Disorder (GAD-2) and Patient-Health-Questionnaire (PHQ-2) measures. To assess how such symptoms and other factors (e.g., loneliness) had changed, we asked to what extent these occurred before and since the lockdown. We included 965 fully completed questionnaires in the analysis. The proportions of participants screening positive for depression and anxiety were significantly higher during the lockdown than before the lockdown, based on their reported symptoms for these periods: 29.3%% vs. 13.5% (*p* < 0.001), and 37.1% vs. 25.7% (*p* < 0.001) respectively. Lonely and young participants were more likely to acquire depression. About one in five participants reported suicidal ideation. Our findings suggest that the COVID-19 pandemic has exacerbated already existing mental health disparities between sexual minority adults and the general population. These exacerbations may be the result of increased loneliness and social isolation. The results highlight the need for stimulating and strengthening social connectedness within the LGBTQI community during and in the aftermath of the COVID-19 pandemic, and the need for maintaining mental health services for such groups during pandemic restrictions.

## Introduction

The public health threat of the COVID-19 pandemic required unprecedented measures to limit the spread of infections (1). Many countries imposed guidelines and measures to reduce real-life human interaction, in particular during the first wave of infections (2). The measures issued by the Belgian government included restricting real-life social contact to household members, and keeping a distance of 1.5 meters from other persons (i.e., “physical distancing”) from March 18 to May 10, 2020 (3, 4). The implications of such restricted social contact are not fully understood. Worsening of mental health problems have been related to loneliness during the COVID-19 crisis (5). Increasing evidence shows that the COVID-19 pandemic had negative effects on mental health, such as increased anxiety or depression in the general population (6).

Lesbian, gay, transgender, queer, intersex and other sexual and gender minorities (hereafter referred to as “LGBTQI”) are at higher risk for adverse mental health outcomes such as suicidal ideation, depression and feeling lonely (7–11). A systematic review estimates lifetime prevalence of suicide attempts in this population to be four times higher than in the general population, and depression 1.5 times higher (12). These mental health disparities may be the result of minority stigma and discrimination (7, 13). Social embeddedness and social support by peers or family can be crucial to counteract such mental health disparities (7–11). COVID-19 measures, such as physical distancing and stay-at-home requirements typically allowed for real-life social contact between household members only. LGBTQI persons are less likely to be married, or to have children, increasing the risk for loneliness during the COVID-19 crisis (9). This may imply negative consequences of COVID-19 related measures on their mental health.

The objective of this analysis was to examine changes in relevant mental health indicators, i.e., depression, anxiety and suicidal ideation among Belgian LGBTQI persons before and during the first weeks of the first COVID-19 lockdown. We explored which sociodemographic, behavioral and social factors were associated with acquired depression or remission. Such insights are crucial to understand the impact of the COVID-19 lockdown measures on this population (14).

## Materials and Methods

### Design and Data Collection

We analyzed cross-sectional data from an online survey. The questionnaire was available in Dutch, French and English between April 10 and April 27, 2020, i.e., during the first Belgian lockdown (4). It was disseminated by sexual health organizations and organizations for LGBTQI persons. Inclusion criteria were: being 18 years or older, not exclusively heterosexual and born or living in Belgium. Eligible participants provided informed consent by agreeing to participate, after having been informed about the study and its procedures. There was one round of data collection. To assess how certain factors (e.g., loneliness) or health outcomes (e.g., depression) had changed during the lockdown, we asked to what extent these occurred before and since the lockdown. Detailed data collection procedures were published elsewhere (4).

### Measures

We used two self-reported screening measures for anxiety and depression: the “Generalized Anxiety Disorder 2-item” (GAD-2) and “Patient Health Questionnaire 2-item” (PHQ-2) respectively. The scales assess the frequency of related symptoms, with answering options ranging from 0 (never or not at all) to 3 (almost every day) (15). We considered an individual to screen positive for anxiety or depression when the sum of both items was three or higher on GAD-2 or PHQ-2 respectively, a score requiring further clinical evaluation (15). Suicidal ideation was evaluated by assessing the frequency of PHQ-9 item “thoughts that you would be better off dead or hurting yourself”, with the same answering options. It was dichotomized so as 1 denoted having these thoughts at least some of the time and 0 none. Within the questionnaire, participants first indicated to what extent these symptoms (i.e., depression, anxiety and suicidal ideation) occurred *before* the lockdown, and subsequently *since* the lockdown period.

The questionnaire also included questions on sociodemographic characteristics, sexual orientation, partner and living situation, migration status and HIV status at the time of data collection. We assessed whether drug use, alcohol abuse or financial hardship was present before the lockdown. Within the questionnaire, we measured loneliness before and since the lockdown with the three-item UCLA loneliness scale (16), and used answering options ranging from 1 (never or not at all) to 4 (almost every day), in line with the other scales. Scores 8 or higher were coded as “lonely” and below 8 as “not lonely”, based on the cut-off score 47 of the 20-item measure (17). We asked about social contact in different types of relationships before and since the COVID-19 lockdown.

### Analysis

There may be various reasons for not having fully completed the questionnaire, e.g., having clicked the link to view the questionnaire or parts thereof, having lost internet connection, or decreased motivation to participate. We explored associations between age, gender and region, and questionnaire completeness using chi^2^ tests among those who had agreed to participate to explore potential bias. We only included fully completed questionnaires in the analyses of this study. We used McNemar's test to assess significant differences (p < 0.05, asymptotic) between the two time periods for the dichotomized depression, anxiety and suicidal ideation variables. Because of the substantial increase found in depressive symptoms, we additionally explored risk factors for potentially acquired depression (i.e., screening negative before and positive for depression during the lockdown), and for depression remission (i.e., screening positive before and negative during the lockdown). To explore to what extent risk factors differed compared to the time before COVID-19 we also examined factors associated with screening positive for depression before the lockdown. For the latter, we calculated odds ratios (OR) and 95% confidence intervals (95% CI) using logistic regression. Self-reported social contact and loneliness before the lockdown were used to calculate the associations with screening positive for depression before the lockdown, whereas the variables relating to the lockdown period were used to calculate the odds of acquiring depression or remission. We used SPSS version 27 for the analysis.

## Results

### The Sample

**Figure 1:**
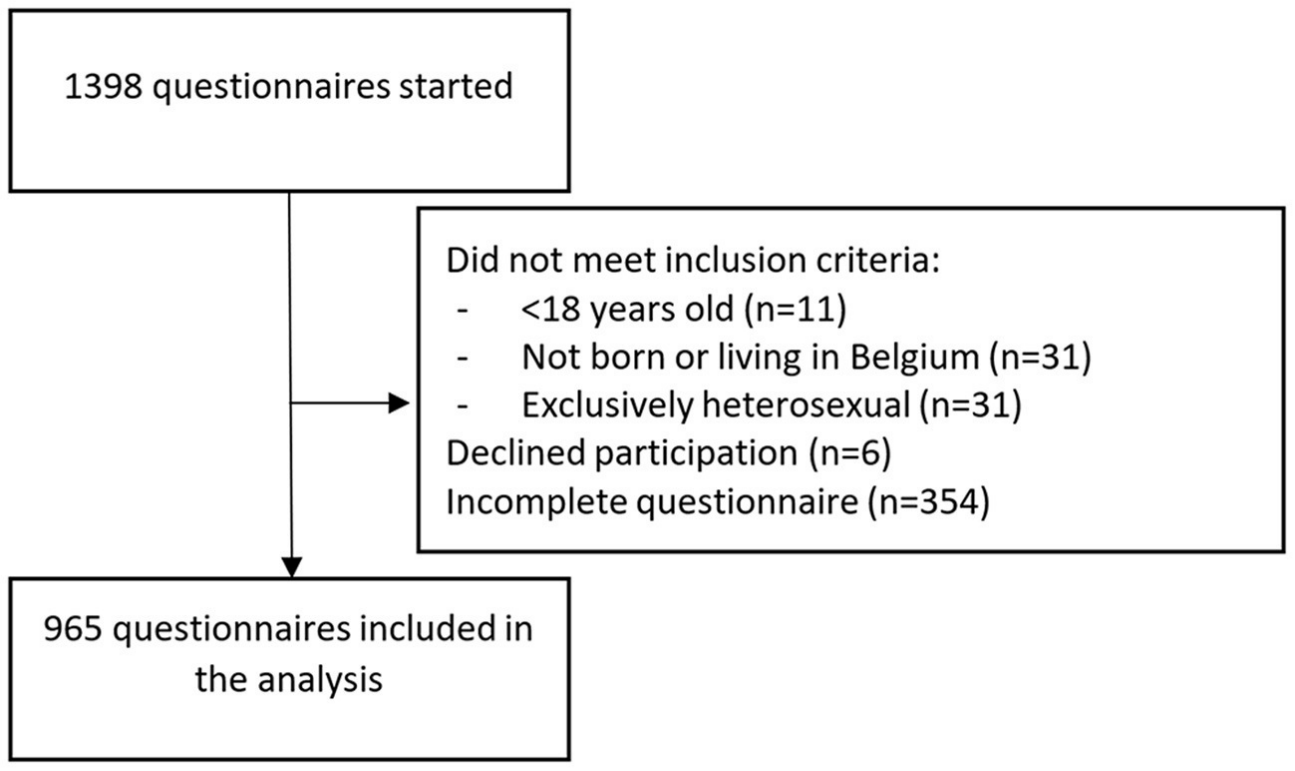
Sample selection.

Among the 1,398 started questionnaires, 103 had missing data on inclusion criteria or the consent procedure, 73 did not meet the inclusion criteria, 6 declined participation and 251 were incomplete. Questionnaire incompleteness was more likely among participants living in Wallonia (31.3%) than participants living in Flanders (20.4%) or Brussels (16.9%, *p* = 0.004). We included 965 fully completed questionnaires into the analysis (see Figure 1). Almost half of the respondents (46.6%) were between 36 and 55 years old (Table 1). The majority reported a male gender (72.5%), being born in Belgium (86.3%), living in Flanders (68.1%, the Northern, Dutch-speaking region), and being HIV negative (86.1%). Participants reported having had less real-life social contact with gay friends, heterosexual friends, family and colleagues during the lockdown than before„ and more online only social contact.

**Table 1:** Bivariate analyses for associations between sociodemographic factors, alcohol and drug use and social contact, and having or gaining a positive screening status for depression before and during the lockdown.

**Sociodemographic characteristics**	**Total sample (N=965)**		**Bivariate analyses for screening positive for depression before lockdown**		**Bivariate analyses for acquired depression during lockdown^e^**	
	N (%)		**OR (CI 95%)**	***p*** **-value**	**OR (CI 95%)**	***p*** **-value**
Age				0.001		<0.001
18–35 years	422 (43.7)		Ref.		Ref.	
36–55 years	450 (46.6)		0.46 (0.31–0.69)		0.55 (0.39–0.77)	
56 years or higher	93 (9.6)		0.60 (0.31–1.18)		0.16 (0.06–0.40)	
Gender				<0.001		0.205
Male	700 (72.5)		Ref.		Ref.	
Female	162 (16.8)		1.41 (0.87–2.30)		1.48 (0.97–2.27)	
Transman or -woman	33 (3.4)		5.04 (2.41–10.52)		1.33 (0.48–3.74)	
Other	70 (7.3)		1.60 (0.83–3.11)		1.52 (0.83–2.80)	
Born in Belgium	833 (86.3)		0.62 (0.38–1.00)	0.049	0.76 (0.48–1.21)	0.242
Region^a^				0.034		0.506
Flanders	648 (68.1)		Ref.		Ref.	
Brussels region	216 (22.7)		1.34 (0.86–2.08)		1.06 (0.71–1.59)	
Wallonia	88 (9.2)		2.04 (1.17–3.58)		1.40 (0.80–2.46)	
At least one steady partner	560 (58.0)		0.57 (0.40–0.83)	0.003	0.58 (0.42–0.81)	0.001
Financial hardship^b^	70 (7.4)		2.67 (1.52–4.69)	0.001	2.51 (1.39–4.52)	0.002
Excl. homosexual identity	675 (69.9)		0.46 (0.32–0.67)	<0.001	0.68 (0.48–0.96)	0.030
HIV positive status^c^	127 (13.9)		0.84 (0.47–1.50)	0.562	1.10 (0.68–1.78)	0.702
***Drug & alcohol use (before)***					
Drug use	296 (30.7)		1.05 (0.70–1.56)	0.818	1.35 (0.96–1.92)	0.087
Alcohol abuse	92 (11.9)		1.79 (1.04–3.07)	0.036	1.10 (0.62–1.94)	0.747
***Social contact and loneliness***	*Before*	*Since*				
Gay friends				0.005		0.797
Real-life contact^d^	774 (80.2)	254 (26.3)	Ref.		Ref.	
No contact	76 (7.9)	157 (16.3)	1.55 (0.82–2.93)		1.19 (0.71–2.00)	
Online contact only	115 (11.9)	554 (57.4)	2.19 (1.35–3.57)		1.06 (0.72–1.56)	
Hetero friends				0.004		0.476
Real-life contact^d^	789 (81.8)	215 (22.3)	Ref.		Ref.	
No contact	60 (6.2)	162 (16.8)	1.31 (0.62–2.74)		1.39 (0.81–2.37)	
Online contact only	116 (12.0)	588 (60.9)	2.24 (1.39–3.63)		1.20 (0.79–1.83)	
Family				0.025		0.113
Real-life contact^d^	740 (76.7)	258 (26.7)	Ref.		Ref.	
No contact	75 (7.8)	153 (15.9)	2.14 (1.20–3.85)		1.73 (1.02–2.93)	
Online contact only	150 (15.5)	554 (57.4)	1.39 (0.85–2.27)		1.37 (0.91–2.05)	
Colleagues				<0.001		0.325
Real-life contact^d^	735 (76.2)	250 (25.9)	Ref.		Ref.	
No contact	159 (16.5)	263 (27.3)	2.72 (1.76–4.21)		1.37 (0.86–2.17)	
Online contact only	71 (7.4)	452 (46.8)	2.52 (1.38–4.62)		1.32 (0.88–1.98)	
Lonely	110 (11.4)	356 (36.9)	10.95 (7.03–17.05)	<0.001	9.95 (6.82–14.50)	<0.001

*Excl.= exclusively; Ref.=Reference category; OR = odds ratios; CI: confidence interval*.

a *“not living in Belgium” (n = 13) excluded from analysis for this variable*.

b *“prefer not to say” (n = 14) excluded from analysis for this variable; Proportion indicating to be struggling with their income before 18 March*.

c *“prefer not to say (n = 5) and “don't know” (n = 45) excluded from analysis for this variable*.

d *“Real life contact” can also include “online contact”, unless it was strictly online only*.

e *Bivariate analyses among participants who did not have depression before the lockdown (n = 835)*.

### Depression, Anxiety and Suicidal Ideation

**Figure 2:**
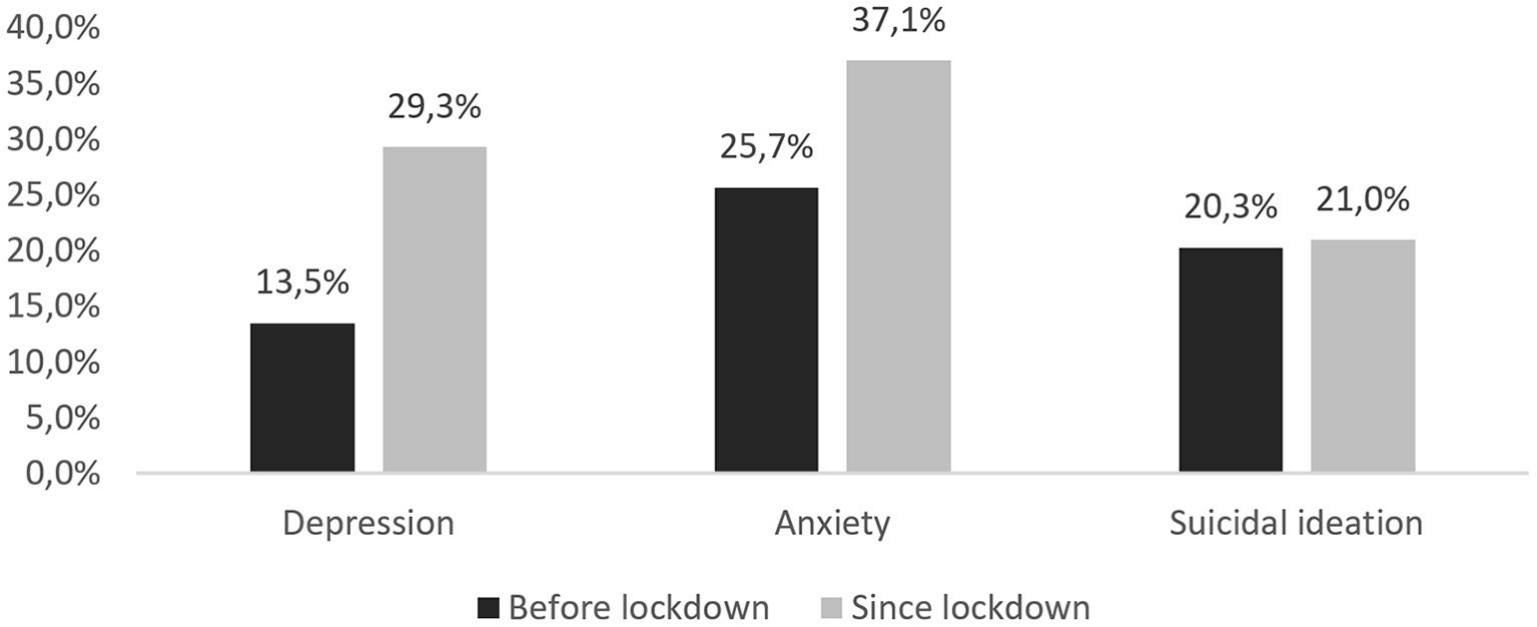
Proportions of participants screening positive for depression, anxiety and suicidal ideation before and since lockdown (*N* = 965).

About 29.3% of the participants screened positive for depression based on their symptoms reported for the period since the lockdown (see Figure 2), whereas this was significantly lower for the period before (13.5%, *p* < 0.001). Likewise, the proportion of participants screening positive for anxiety was significantly higher (37.1% ) based on symptoms reported for the period since the lockdown, compared to before (25.7%, *p* < 0.001). There were no significant changes in the proportion of participants with suicidal ideation (*p* = 0.510). About one in five participants reported having thoughts of being better off dead or hurting oneself at least some of the time since the lockdown (i.e., suicidal ideation).

### Factors Associated With Acquired Depression or Remission During the Lockdown

Among participants who did not screen positive for depression before the lockdown (*n* = 835), those who were younger than 36 years, had no steady partner, struggled financially, not identified as exclusively homosexual and reported feeling lonely were significantly more likely to have acquired depression during the lockdown, when compared with their counterparts (Table 1).

All factors associated with acquired depression during the lockdown were also significantly associated with screening positive for depression before the lockdown. Identifying as a transman or -woman, being born outside of Belgium, living in Wallonia (i.e., the Southern, French-speaking region), alcohol abuse before the lockdown, having only online social contact with gay or hetero friends or colleagues (vs. real-life contact), or having no social contact with family or colleagues before the lockdown were associated with screening positive for depression before, but not with acquiring depression during the lockdown.

Among 130 participants who screened positive for depression before the lockdown, 29 (22.3%) no longer did so during the lockdown. Being lonely during the lockdown was the only variable that significantly increased the likelihood for screening positive for depression during the lockdown, among those who screened positive before (OR: 4.95; 95%CI: 2.05–11.95).

## Discussion

In our sample of LGBTQI individuals we found a substantially higher proportion of participants screening positive for depression and anxiety during the first COVID-19 lockdown, when compared with their symptoms reported for the period before. The increase in the proportion of persons screening positive for depression (from 13.5 to 29.3%) was more substantial than in the general Belgian population (from 9.5 to 20%, using the PHQ-9 measure) (18). It was also higher than what was found in other studies among the general population during the early phase of COVID-19 (between March and May 2020), e.g., in Germany (14.3%), Spain (18.7%) and the USA (23.5%) (19–21). A study among LGBTQI persons in Hong Kong and an international online study among men who have sex with men (MSM) found similar proportions of depression (31.5% and 35%), using the same PHQ-2 measure in this period (22, 23). Similarly, the proportion of individuals screening positive for anxiety during the lockdown (37.1%) was higher than in the general population in Belgium (23%), Spain (21.6%) and the USA (30.8%); it was comparable to MSM in an international online study (34%), but higher than among LGBTQI persons in Hong Kong (27.9%) (19–23). Given the higher increase in proportions screening positive for depression and anxiety when compared to the general population, it can be suggested that the COVID-19 pandemic has exacerbated already existing mental health disparities between LGBTQI and the general population. This is in line with a recent Swiss study demonstrating that the COVID-19 crisis had a greater psychological impact on sexual minority men compared to heterosexual men (24).

Having real-life social contact with gay friends, heterosexual friends and colleagues was associated with a lower likelihood of screening positive for depression before the lockdown. We found that such social contact had generally become online only, but was not associated with acquiring depression during COVID-19 lockdown. Younger LGBTQI persons were more likely to acquire depression. This may be due to being isolated with unsupportive families or being unable to meet like-minded peers (25, 26). Participants feeling lonely during the lockdown and those without a steady partner were more likely to have acquired depression. The association between increased loneliness early in the COVID-19 pandemic and a higher likelihood of acquiring depression was also found in the USA (17). Hence, our findings suggest that a sub-group of LGBTQI persons may be at higher risk to become socially isolated during a pandemic with restricted real-life social contact, and that such increased loneliness can have detrimental effects on their mental health.

The increase in adverse mental health outcomes may be due to the reduced connections with the LGBTQI community and fewer possibilities for meeting like-minded persons (22, 27). When real-life social contact is restricted, stimulating online contact within vulnerable communities such as LGBTQI may be a crucial strategy to prevent social isolation among people vulnerable for loneliness (e.g., single persons, or young LGBTQI living with their parents). When implementing measures to restrict physical contact to limit the spread of infections, it may be recommended to allow for social contact beyond traditional households for those who are at higher risk of becoming socially isolated. LGBTQI organizations should be supported to strengthen community connectedness and stimulating social support among their members during next waves of infections and in the aftermath of COVID-19. Further studies should investigate to what extent COVID-19 related mental health impact reverses after lifting of physical distancing measures.

In our study, about one in five participants reported thoughts of being better off dead or hurting oneself (i.e., suicidal ideation) at least some of the time during the first COVID-19 lockdown. Although this did not differ significantly from the period before, it remains alarmingly high. Suicidal ideation and behaviors present an important public health problem in Belgium with 4.3% of the population seriously considering suicide in the last 12 months and 0.2% having attempted to commit suicide in 2018 (28). Our findings highlight the urgent need to strengthen LGBTQI community support in addressing this mental health problem within a comprehensive suicide prevention strategy.

### Limitations

These findings are of limited generalizability due to the potential self-selection bias inherent to online surveys. In the questionnaire, we asked about depression, anxiety, suicidal ideation, social contact and loneliness *before* and *during* the lockdown. Recall bias cannot be ruled out, in particular for questions pertaining to the period before COVID-19. The majority were MSM, which may not be representative of the LGBTQI community. Participants living in Wallonia were less likely to complete the questionnaire, but we have no further indication as to how this may have impacted on the results. We used brief self-reported screening measures, which only gives an indication requiring further evaluation for clinical diagnosis. Using more specific measures may have yielded different results.

## Conclusion

The proportion of LGBTQI persons who should be further evaluated for depression and anxiety had increased significantly during the early phase of COVID-19. These exacerbations may be the result of increased loneliness and social isolation. About one in five participants reported suicidal ideation, requiring specifically targeted interventions. Our findings highlight the need for stimulating and strengthening social connectedness within the LGBTQI community during and in the aftermath of the COVID-19 pandemic, and the need for maintaining mental health services during pandemic restrictions.

## Data Availability Statement

The datasets presented in this article are not readily available because of ethical restrictions. Requests to access the datasets should be directed to treyniers@itg.be.

## Ethics Statement

The studies involving human participants were reviewed and approved by Institutiontal Review Board of the Institute of Tropical Medicine. The patients/participants provided their informed consent to participate in this study in the online questionnaire.

## Author Contributions

TR, VB, ET, BV, CN, and EW were involved in setting up the study and data collection. TR, VB, CN, and EW contributed to the data analysis. TR drafted the manuscript, and all authors commented on subsequent versions. All authors approved the final manuscript. All authors contributed to the article and approved the submitted version.

## Funding

TR is a postdoctoral fellow of the Research Foundation—Flanders. The study consortium received funding from the Research Foundation—Flanders as an SBO-project (S004919N).

## Conflict of Interest

The authors declare that the research was conducted in the absence of any commercial or financial relationships that could be construed as a potential conflict of interest.

## Publisher's Note

All claims expressed in this article are solely those of the authors and do not necessarily represent those of their affiliated organizations, or those of the publisher, the editors and the reviewers. Any product that may be evaluated in this article, or claim that may be made by its manufacturer, is not guaranteed or endorsed by the publisher.
